# Comprehensive analysis of landslide stability and related countermeasures: a case study of the Lanmuxi landslide in China

**DOI:** 10.1038/s41598-019-48934-3

**Published:** 2019-08-27

**Authors:** Zheng Han, Bin Su, Yange Li, Yangfan Ma, Weidong Wang, Guangqi Chen

**Affiliations:** 1 School of Civil Engineering, Central South University, Changsha, Hunan 410075 China; 2 State Key Laboratory of Geohazard Prevention and Geo-environment Protection, Chengdu University of Technology, Chengdu, Sichuan 610059 China; 3 Key Laboratory of Heavy-haul Railway, Ministry of Education, Changsha, Hunan 410075 China; 4 Department of Civil and Structural Engineering, Kyushu University, 819-0395 Fukuoka, Japan

**Keywords:** Natural hazards, Geomorphology

## Abstract

We report on a comprehensive method for analyzing landslide stability and the mitigation effect of countermeasures in this paper. The proposed method is a combination of theoretical method and numerical method. To address the uncertainties of the soil strength parameters, the rational values of these parameters are comprehensively determined by the back-analysis result of the reliability method and the result by the quantitative method, as well as the *in-situ* geological test. To evaluate the slope stability, the limit analysis using the 2D upper bound method and the FEM simulation using strength reduction method are performed, respectively. In order to illustrate the presented method, the so-called Lanmuxi landslide in China is selected as a case study. Results demonstrated that the stress and strain majorly concentrated at the toe and crown of the slope. According to the analysis results, countermeasures consisting of anchor lattice beams, landslide piles, and cracks filling, are suggested to reduce the failure risk of the landslide. Effect assessment based on the FEM analysis verifies the feasibility and effectiveness of the recommended countermeasures.

## Introduction

Landslides are a common geological phenomenon in mountainous regions worldwide, posing a severe risk to local infrastructures. During the years from 2004 to 2010, 2620 fatal landslides in total were recorded, causing 32,322 fatalities^1^. To protect residents and infrastructures against landslides, a rational design of countermeasures is necessary. The commonly used countermeasures include supporting measures and drainage measures^2^. Current studies in landslide hazard mitigation mainly focus on the overall design principles, structure optimization, and the mitigation effect evaluation. However, it is widely accepted that an effective countermeasure against landslide depends on the deep understanding of landslide mechanisms and rational analysis of landslide stability, which remain a scientific challenge.

Up-to-date studies on landslides can be briefly summarized in several categories, e.g., landslide mechanisms^3–5^, failure behaviour simulation^6–9^, sensitivity analysis^10–12^, as well as countermeasure design and optimization^13,14^. These studies have underlaid a solid theoretical foundation for landslide mitigation work.

The studies on the landslide mechanisms provide a fundamental understanding of the landslide process. Previous studies^15–17^ have long proposed various failure models of landslides. These remarkable studies also substantiated the key factors dominating the landslide process, e.g., topography, material strength^3,18^, as well as some other triggering factors, e.g., rainfall precipitation and earthquakes^19,20^. These studies explore important features of landslide process and benefit the practical work.

Most studies regarding landslide mechanisms are commonly empirically or semi-empirically based. However, a physical and quantitative analysis of the landslide process is required, because slope stability, as well as magnitude and possible runout extension are the key parameters to the countermeasure design. In this context, some attempts have been made to evaluate slope stability. In order to reproduce the landslides process, it is essential to determine the key parameters of landslides, cohesion strength *c*, and internal friction angle *φ*. The two parameters are mainly based on the *in-situ* test, engineering experience analogy, and back analysis^21^ at present. Being an efficient solution to determine the key parameters, the back-analysis method can be briefly summarized into two categories, the deterministic method^22,23^ and reliability method^24–28^. Previous studies^22^ also support that the back-analysis provides reliable results approximating the expected shear strength parameters.

In order to estimate the potential risk of slope failure, the potential magnitude and runout extension should be estimated. It is a major issue and a complex task for landslide mitigation work. Numerical simulation provides an alternative solution for this purpose. Several models have been developed and applied to practical work. They use the discontinuous deformation analysis (DDA) method^29–31^, the finite element method (FEM)^32,33^, the discrete element method (DEM)^34,35^, the smoothed particle hydrodynamics method (SPH)^7,9^, to analyse the stability and simulate the failure process of the landslide. Some other studies also incorporate hydraulically based models to simulate landslide behaviour, such as DDA-SPH coupled method^36^ and shallow water assumption-based model^37^. However, difficulties remain in the measurement of related parameters in these numerical models. Owing to the significant individual differences from case to case, as well as the temporal and spatial variation, some important parameters are difficult to determine, requiring trial-and-error adjusting during simulation^38^. In this sense, a comprehensive analysis of landslide stability and failure process simulation remains a major topic.

In this paper, we report on a comprehensive method for analysing landslide stability and its failure process. Two essential issues are discussed, i.e., comprehensive estimation of soil strength parameters using the back-analysis results using the reliability method, and the evaluation of the slope stability using the limit analysis and the FEM simulation. The so-called Lanmuxi landslide in China is selected as a case study to illustrate the presented method.

## Methods

### Back analysis of key parameters

In this paper, we first use reliability theory to back analyse the shearing strength parameters. Back analysis is an effective method for determining landslide parameters. For landslide mitigation work, the safety factor *F*_*s*_ is commonly used as an indicator for the stability. The slope is likely to fail when *F*_*s*_ ≤ 1, while is under a limit state when *F*_*s*_ = 1. In the reliability method, the function equation is incorporated to represent the limit state of the slope. The function is as below,

1 $$Z={\rm{g}}({X}_{1},{X}_{2},{X}_{3},\ldots ,{X}_{n})={F}_{s}-1$$

where *X*_1_, *X*_2_, *X*_3_, …, *X*_*n*_ denotes random variables regarding to slope stability. In our study, only two parameters, the cohesion *c* and friction angle *φ* are considered and assumed as random variables. Slope stability can be quantitated by the value of *Z*. *Z* < 0 denotes that the slope is at risk of failure, while *Z* = 0 represents that the slope stays in the limit state. When *Z* > 0, the slope keeps stable. Another key parameter in reliability theory is the slope reliability index *β*, which refers to the probability of a slope that completes the pre-determined function under the specified condition and time. Based on previous studies^39^, the reliability index *β* can be expressed as:

2a $$\beta =\sqrt{\widehat{{X}_{1}^{2}}+\widehat{{X}_{2}^{2}}+\ldots +\widehat{{X}_{n}^{2}}}=\sqrt{\mathop{\sum }\limits_{i=1}^{n}\widehat{{X}_{i}^{2}}}$$

2b $$\widehat{{{\rm{X}}}_{i}}=\frac{{X}_{i}-{\mu }_{i}}{{\sigma }_{i}},\,(i=1,2,3,\cdots ,n)$$

where *μ*_*i*_ denotes the averaged value, and *σ*_*i*_ represents the standard deviation of the variable. The failure model is established based on the limit state (Z = 0) in the back-analysis of parameters using the reliability method. The final back-analysis result is determined according to the minimum reliability index *β* ^40^, which means, the key parameters *X*_*i*_ are iteratively solved until a minimum *β* is obtained.

The spreadsheet-based method^41,42^ is used to solve the key parameters *X*_*i*_. The reliability index *β* is expressed as the forms of Hasofer-Lind’s reliability index^43^,

3 $$\beta =\mathop{\min }\limits_{x\in F}\sqrt{{(X-\mu )}^{T}{{C}_{x}}^{-1}(X-\mu )}$$

where *X* denotes random variables, *μ* is the averaged value of the random variables, *C*_*x*_ is the covariance matrix of variables, *F* is the failure domain. Equations (1,3) can be iteratively solved according to the optimization algorithm. Compared with the traditional deterministic back analysis method, reliability method generates better results because the effect of the uncertainties in the landslide parameters can be considered.

### Stability analysis based on the 2D upper bound (UB) theory

Presently, the majority of slope stability analyses evaluate a safety factor using a 2D representation of the slope, while 3D analyses of slope stability are much less reported^44,45^. It can be explained in part by the fact that a 3D model is likely to introduce a much larger number of degrees-of-freedom, which demands significantly more computational time and effort than the 2D model^46^. Meanwhile, in the 3D model, the failure surface does not only cross weak soil layers, but also strong ones with uncertainties, consequently increases the calculated factor of safety, e.g., 14% to 18% increasement by Reyes and Parra^47^ and 13.9% by Xie^48^, compared with the minimum factor of safety in the 2D model. In a word, the 2D analysis could lead to more conservative results than the 3D analysis in slope stability problems.

**Figure 1:**
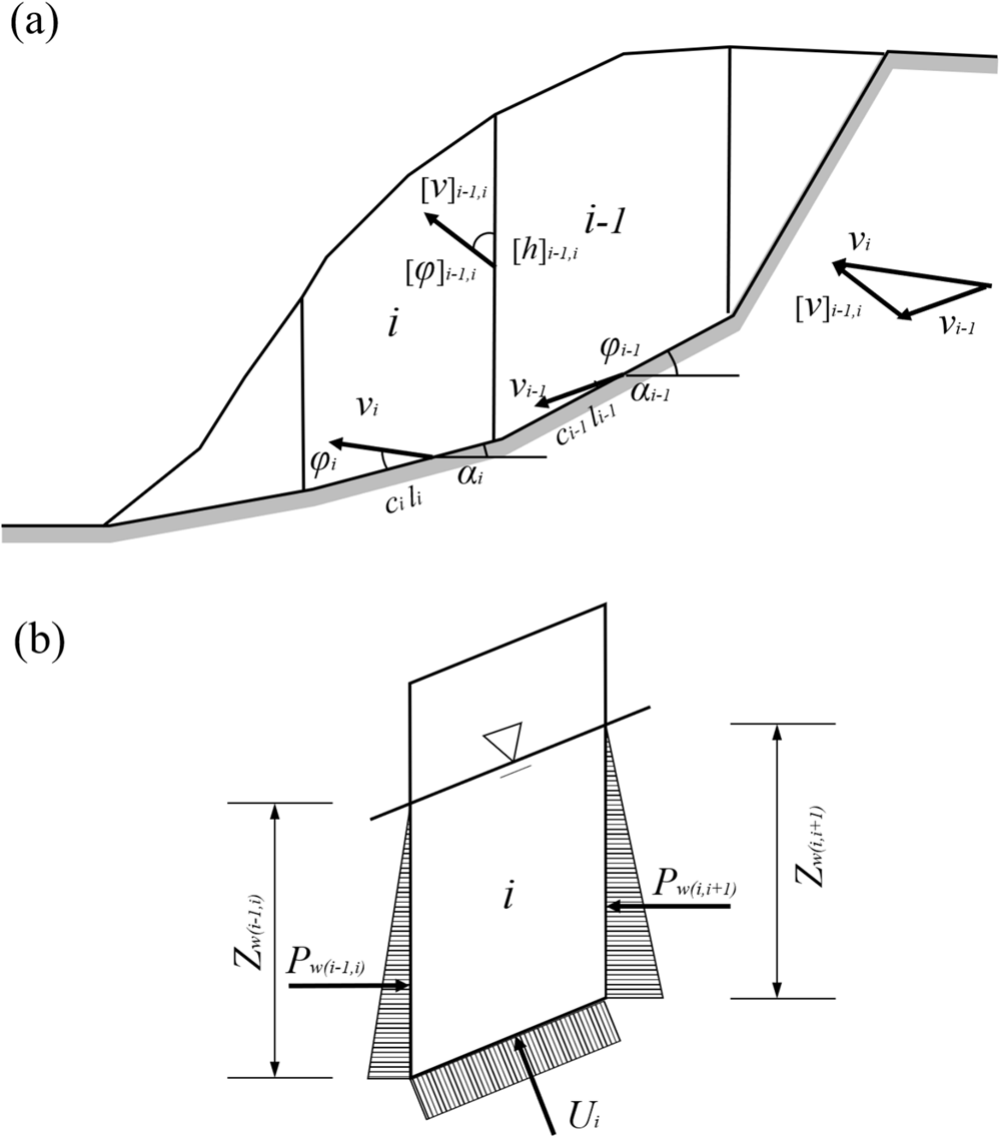
The 2D stability analysis based on the UB theory. (**a**) Virtual velocity field of slope divided into vertical slices; (**b**) Pore water pressure on the sliding layer.

A primary concern in the practice of landslide mitigation is safety. For this consideration, we use the 2D upper bound (UB) limit analysis (LA) method to evaluate a conservative slope stability in this paper. The UB method is an energy-based method, using the principle of virtual work^49,50^. According to the virtual work equations, the internal work of the slope should be equal to its external work (i.e., *D*_*int*_ = *W*_*ext*_) in the limit state. A kinematically admissible velocity field^51^ is presented for calculating the virtual work. The velocity formula can be described as follows,

4 $${v}_{i}={v}_{i-1}\frac{\cos ({\alpha }_{i-1}-{\phi }_{i-1}-{[\phi ]}_{i-1,i})}{\cos ({\phi }_{i}+{[\phi ]}_{i-1,i}-{\alpha }_{i})}$$

5 $${[v]}_{i-1,i}={v}_{i}\frac{\sin ({\phi }_{i}-{\phi }_{i-1}-{\alpha }_{i}+{\alpha }_{i-1})}{\cos ({\alpha }_{i-1}-{\phi }_{i-1}-{[\phi ]}_{i-1,i})}$$

where *v*_*i*_ and *v*_*i*−1_ are the virtual velocities of a slice on the sliding surface. [*v*]_*i*−1,*i*_ represents the relative velocity that defined as the vector difference from *v*_*i*_ to *v*_*i*−1_. It should be noticed that *v*_*i*_, *v*_*i*−1_ and [*v*]_*i*−1,*i*_ satisfy the closure relations. *φ*_*i*_ and *φ*_*i−*1_ are the internal friction angles of the neighboured slice, respectively. [*φ*]_*i*−1,*i*_ denotes the relative internal friction angle in the vertical direction of slices. *α*_*i*_ and *α*_*i−*1_ are the inclined angles. Therefore, the UB solution of the safety factor can be calculated by the following equation:

6 $${F}_{s}=\frac{{\sum }_{i=1}^{n}{l}_{i}{v}_{i}ccos{\phi }_{f}+{\sum }_{i=2}^{n}{[h]}_{i-1,i}{[v]}_{i-1,i}ccos{[\phi ]}_{f}}{{\sum }_{i=1}^{n}{W}_{i}{v}_{i}\,\sin ({\alpha }_{i}-{\phi }_{i})+{\sum }_{i=1}^{n}{U}_{i}{v}_{i}sin{\phi }_{f}+{\sum }_{i=2}^{n}{[v]}_{i-1,i}{P}_{w(i-1,i)}sin{[\phi ]}_{f}}$$

where *φ*_*f*_ = arctan(tan*φ/F*_*s*_). *l*_*i*_ is the sliding surface length of the slice *i*. *P*_*w*_ and *U* represent the external pressure arisen by pore water. *P*_*w*_ can be calculated by ${P}_{w}=\frac{1}{2}{\rho }_{w}{Z}_{w}$, where *P*_*w*_ denotes the density of water and the Z_*w*_ represents the depth of pore water. *U* is obtained by constructing the equilibrium equation with *W*_*i*_ and *α*_*i*_, at vertical direction. *W*_*i*_ is the gravity of the slice *i*. [*h*]_i−1,*i*_ is the length of the interface between the slice *i* and the slice *i-*1. Equations (4–6) can be further explained by Fig. 1.

### Stability analysis using the FEM strength reduction method

Compared with the traditional LA method, the finite element method (FEM) performs better with the advantage of considering much complex boundary conditions, as well as the non-homogeneity of the soil and rock mass. Another advantage of the FEM method is that the stress and deformation field can be also obtained. The FEM strength reduction method has been proposed and applied in the analysis of slope stability^52,53^. It regarded the safety factor *F*_*S*_ as the reduction degree of shear strength of the soil material when the slope reaches the limit state. The safety factor *F*_*S*_ can be redefined as *F*_*S*_ = *c*/*c*_*f*_ or *F*_*S*_ = tan*φ*/tan*φ*_*f*_, where *c* and *φ* are the initially-input shear strength parameters, while *c*_*f*_ and *φ*_*f*_ are the output shear strength parameters in the limit state after reduction, respectively.

The definition of the safety factor mentioned above is consistent with the definition introduced by Bishop^54^, that the safety factor *F*_*S*_ is expressed as bellow,

7 $${F}_{s}=\frac{{\tau }_{f}}{\tau }=\frac{{\int }_{0}^{l}(c+\sigma tan\phi )dl}{{\int }_{0}^{l}\tau dl}$$

where, *τ*_*f*_ represents the shear strength of the slope and can be calculated by the Mohr-Coulomb model with the cohesion and internal friction angle. *τ* denotes the actual shear stress of slope. Equation (7) can be transformed as follow,

8 $$\frac{{F}_{s}}{{F}_{s}}=\frac{{\int }_{0}^{l}(\frac{c}{{F}_{s}}+\sigma \frac{tan\phi }{{F}_{s}})dl}{{\int }_{0}^{l}\tau dl}=\frac{{\int }_{0}^{l}(c^{\prime} +\sigma tan\phi ^{\prime} )dl}{{\int }_{0}^{l}\tau dl}=1$$

Equation (8) means that the slope will reach the limit state with the *c*′ = *c*/*F*_*s*_ and tan*φ*′ = tan*φ*/*F*_*s*_, which is the same with the definition mentioned above. Therefore, in our study, the reduction equations of shear strength are expressed as bellow, deducing with the assumption of the constant external load,

9 $${\rm{c}}^{\prime} =\frac{c}{R}\,,\,\phi ^{\prime} =\arctan (\frac{\phi }{R})$$

where *R* denotes the reduction coefficient with an initiate value of *R* = 1.0. In each iteration step, *R* increases with the stress and deformation analysis based on FEM implemented. The Mohr-Coulomb model is introduced to describe the constitutive relationship of the landslide mass. Commonly, the vertical boundary of the model is fixed in the horizontal direction, while the bottom boundary is constrained in both horizontal and vertical directions. The iteration of simulation breaks until the slope reaches the limit state. The value of the variable *R* under the limit state is regarded as the safety factor *Fs* for the selected landslide profile.

## Results

### Background of the case study

**Figure 2:**
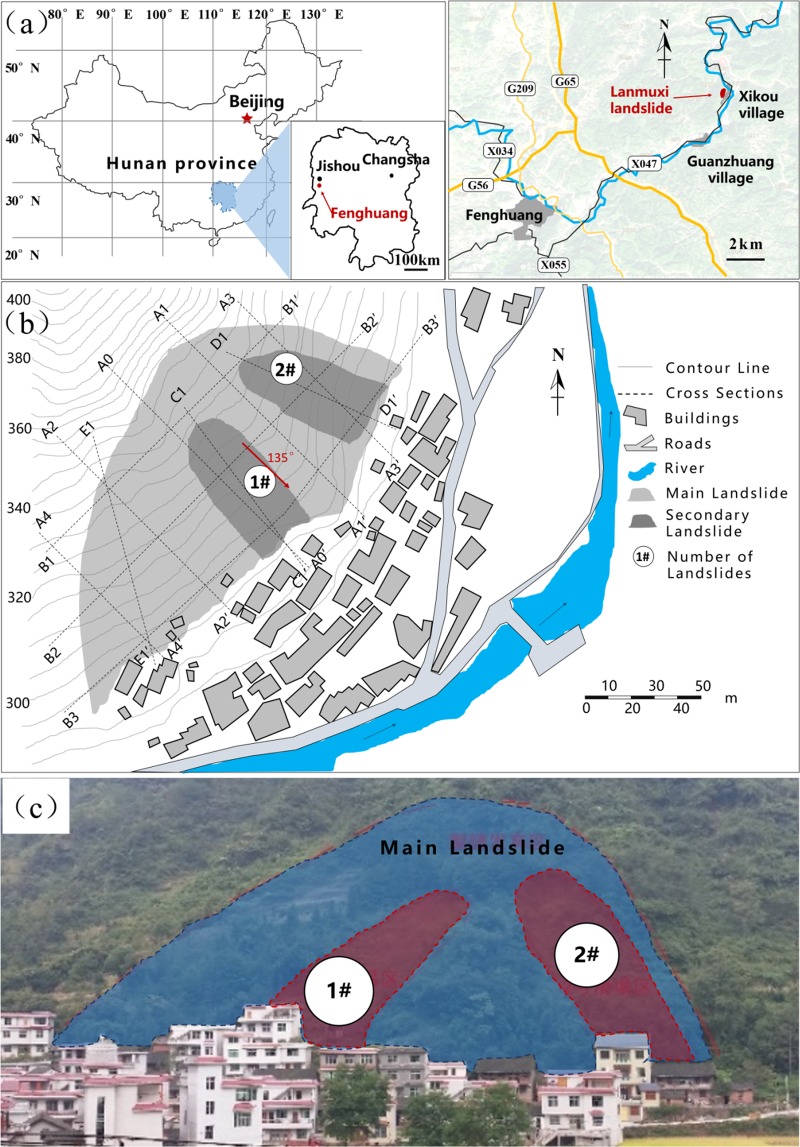
Overview of the Lanmuxi landslide. (**a**) Location of the landslide. (**b**) Map of the Lanmuxi landslide (using Grapher® 10, https://www.goldensoftware.com/products/grapher). (**c**) Photographic view (Photograph was taken by Z. Han).

The so-called Lanmuxi landslide is located in the northwest of Xikou village, Fenghuang town, Hunan province, China (as shown in Fig. 2a). Evidence of previous landslide had been previously observed on July 2014. Owing to the impact of continuous heavy rainfall, the slope deformed, causing cracks in the crown and middle part of the slope body. Subsequently, on September 2014, two secondary landslides were triggered due to a heavy rainstorm, resulting in new cracks occurred in the crown region of the slope, with a maximum width up towards to 0.50 m. The total length of the landslide area is approximately 100.00 m. The maximum width and average thickness of the landslide reach 206.00 m and 4.40 m, respectively. The area of this landslide is estimated 1.37 × 10^4^ m^2^, with a total mass volume of 6.08 × 10^4^ m^3^. The slope body and the sliding layer mainly composed of silty clay, while the substrate consists of argillaceous siltstone. Geological investigation shows that there are only tiny amounts of pore water in the stratum and no groundwater is observed.

The *in-situ* investigation after the secondary landslide event illustrates that the slope is still unstable, threatening 134 residents and 272 local buildings, with a total potential economic loss of 1.8 million dollars. The overview of the Lanmuxi landslide is shown in Fig. 2b,c.

### Back analysis of the shearing strength parameters

In order to describe the state of the landslide under diverse condition reasonably, two different conditions are considered. In Condition 1, the mass of the slope is dry, while in Condition 2, it is supposed to be saturated under the impact of heavy rainfall. The saturated soil mass of the slope increases self-weight and consequently reduces shearing strength, leading to a greater risk for landslide failure.

According to the laboratory test on the three groups of soil material samples that obtained *in-situ*, the mean values of the parameters in this case are listed in Table 1. In the back analysis using the UB theory and the reliability method, the standard deviations are required. The laboratory test shows that the standard deviations of the shear strength parameters in the sliding layer are ± 2.98 kPa and ± 1.04° in the Condition 1, while ± 3.02 kPa and ± 1.96° in the Condition 2. The back analysis based on the deterministic method and reliability method are conducted, respectively. Results are shown in Table 2.

**Table 1:** Parameters of landslide material properties by the laboratory test.

Type	Condition 1: Dry			Condition 2: Saturated			Compression modulus *E*_*s*_ (MPa)	Poisson ratio
	Density *ρ* (g/cm^3^)	Cohesion *c* (kPa)	Frictional angle *φ* (°)	Density *ρ* (g/cm^3^)	Cohesion *c* (kPa)	Frictional angle *φ* (°)		
Main body	1.83	38.17	10.55	1.92	21.17	7.03	5.27	0.32
Sliding layer	1.84	27.67	10.67	1.92	17.33	8.0	5.27	0.32
Substrate	2.48	1.10	37.83	2.58	1.10	37.83	26.21	0.24

**Table 2:** Comparison of the results of two different back-analysis methods.

Method	Condition 1: Dry		Condition 2: Saturated	
	*c*(kPa)	*φ*(°)	*c*(kPa)	*φ*(°)
Deterministic method	22.40	12°	21.63	10°
Reliability method	21.34	10.03	21.21	9.50

Laboratory test and back analysis are comprehensively considered to obtain the parameter of the sliding layer. We use the laboratory test results in Table 1 and the back-analysis results in Table 2 to attain the averaged values of the parameters in Table 3, which are suggested as the best-fitting parameters for stability analysis and FEM simulation.

**Table 3:** Recommended parameters for stability analysis and FEM simulation.

Type	Condition 1: Dry			Condition 2: Saturated			compression modulus *E*_*s*_ (MPa)	Poisson ratio
	Density ρ (g/cm^3^)	Cohesion c (kPa)	Frictional angle Φ (°)	Density ρ (g/cm^3^)	Cohesion c (kPa)	Frictional angle *φ* (°)		
Main body	1.83	38.17	10.55	1.92	21.17	7.03	5.27	0.32
Sliding layer	1.84	23.53*	10.81*	1.92	19.78*	8.99*	5.27	0.32
Substrate	2.48	1.10	37.83	2.58	1.10	37.83	26.21	0.24

*The averaged values in Tables 1 and 2 are used for stability analysis and FEM simulation.

### Stability analysis

As mentioned in the above section, it has been widely accepted that the 2D analysis could lead to more conservative results than the 3D analysis in slope stability problems. For the safety consideration, we evaluate a conservative slope stability using a 2D model. To simplify the actual 3D slope into a 2D model, the length along the plane direction, i.e., C1-C1′ and D1-D1′ in Fig. 2b is hereafter referred to as the two typical slope profiles. Most of the local buildings and infrastructures are distributed at the landslide toes along the directions of the selected profiles. The slopes along the both profiles are presumed to be infinitely wide in the 2D model, negating the 3D effects caused by the infinite width of the sliding mass.

Owing to the significant reduction of the safety factor when soil mass is saturated, we mainly focus on the stability analysis under Condition 2. Two analysis methods, the UB method and the FEM strength reduction method, are conducted, respectively. The results in Table 4 illustrate that the landslide along Profile 2 is unstable (*F*_*s*_ < 1).

**Table 4:** Results of two different stability-analysis methods.

Section	Analyzed safety factors	
	The UB method	The strength reduction method using FEM
Profile 1 (along C_1_-C_1_′)	1.02	1.01
Profile 2 (along D_1_-D_1_′)	0.94	0.89

### Stress and deformation analysis using the FEM method

**Figure 3:**
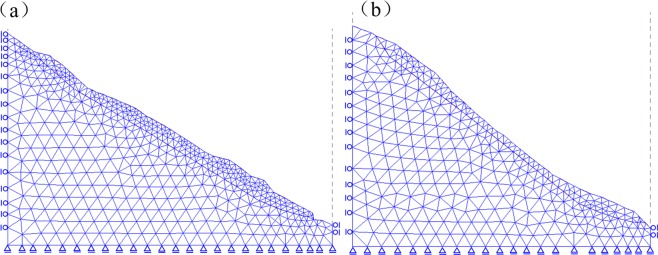
Finite element mesh. (**a**) Profile 1 (C1-C1′). (**b**) Profile 2 (D1-D1′).

In order to perform stress and deformation analysis using the FEM method, the both profiles are meshed into triangle blocks (Fig. 3). Profile 1 is meshed into 1494 blocks with 2515 nodes, while Profile 2 is separated into 1209 blocks with 2029 nodes. In each iteration step of the FEM simulation, stress and deformation are calculated and landslide strength is gradually reduced. Iteration breaks until the slope section comes to the limit state.

**Figure 4:**
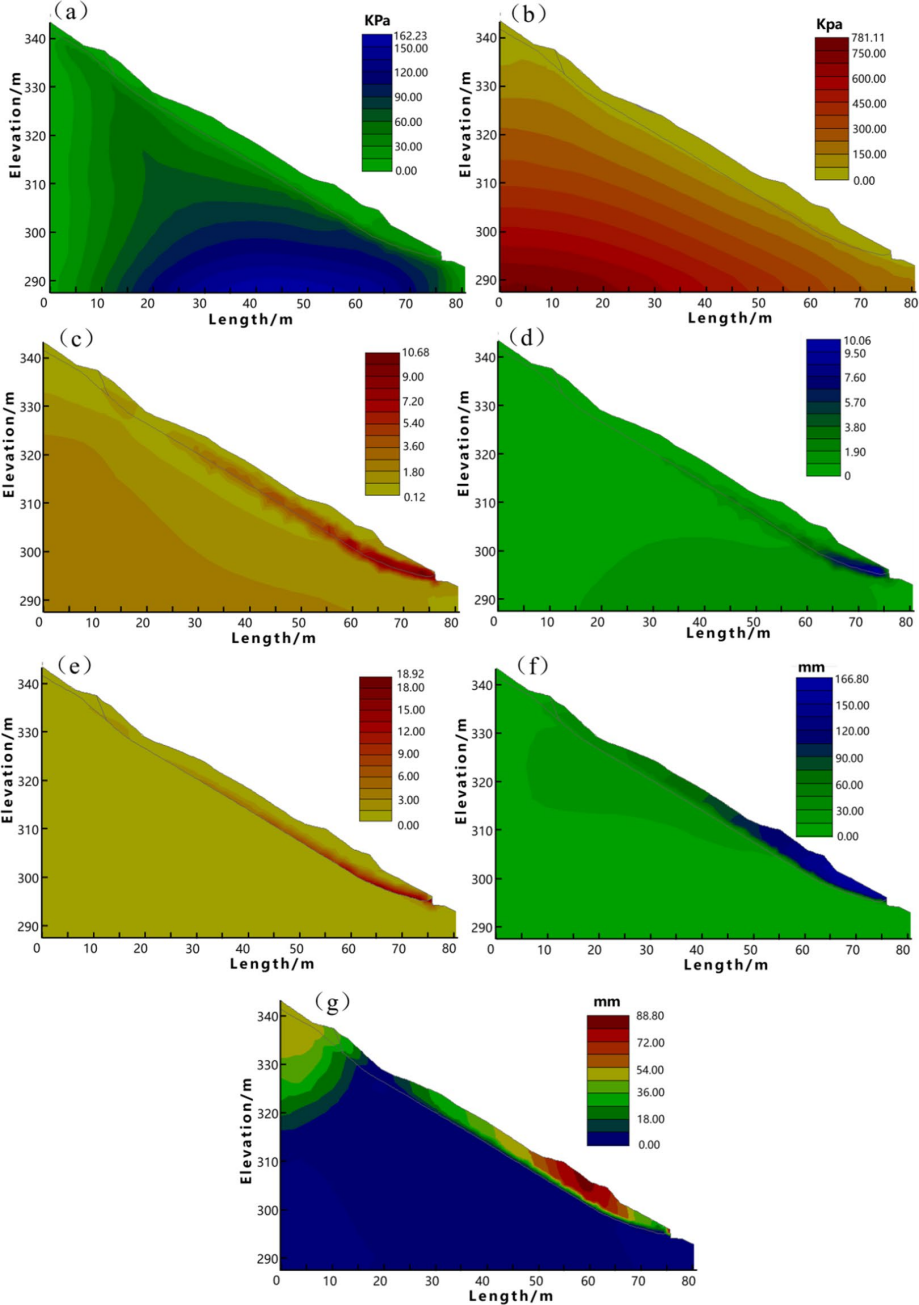
The FEM analysis results of Profile 1. (**a**) Shear stress. (**b**) Effective stress. (**c**) Strain. (**d**) Shear strain. (**e**) Plastic strain (the limit state). (**f**) Deformation in x direction (the limit state). (**g**) Deformation in z direction (the limit state).

**Figure 5:**
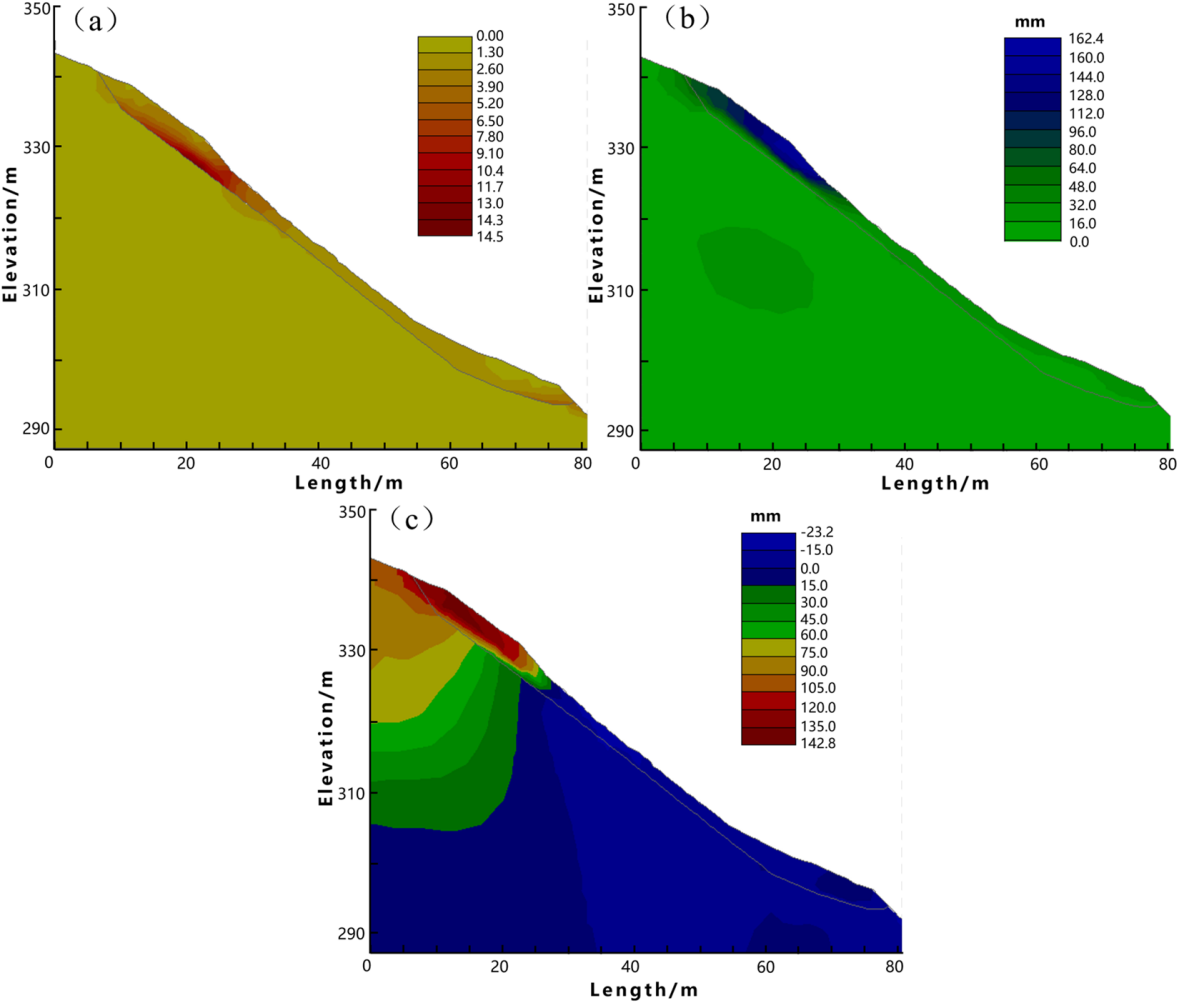
The FEM analysis results of Profile 2. (**a**) Plastic strain (the limit state) (**b**). Deformation in x direction (the limit state) (**c**). Deformation in z direction (the limit state).

Figure 4 and 5 illustrate the stress and deformation distribution. Figure 4a–d demonstrate the distribution of shear stress, effective stress, strain, and shear strain of Profile 1. While the plastic strain, the deformation along both horizontal and vertical directions under the limit state are shown in Fig. 4e–g. In contrast, Fig. 5 only shows the limit state of Profile 2, because the slope along this profile is unstable when soil mass is saturated, limiting the FEM simulation converged. As such, we only use the Eq. (9) to calculate the plastic strain and deformations of this profile in the limit state. Different from the strength reduction method which increases the reduction coefficient *R* as illustrated in Eq. (9), in order to obtain a converged FEM simulation results, we use a decreased *R* in each iteration step in the analysis of Profile 2. The shear strength consequently increases in each step until the unstable slope along Profile 2 reaches a limit state. Figure 4 indicates that the stress and deformation of Profile 1 majorly concentrate at the toe, while Fig. 5 reveals that the stress and strain of Profile 2 in the limit state majorly concentrate at the crown.

## Discussion

### Suggestion of countermeasures

**Figure 6:**
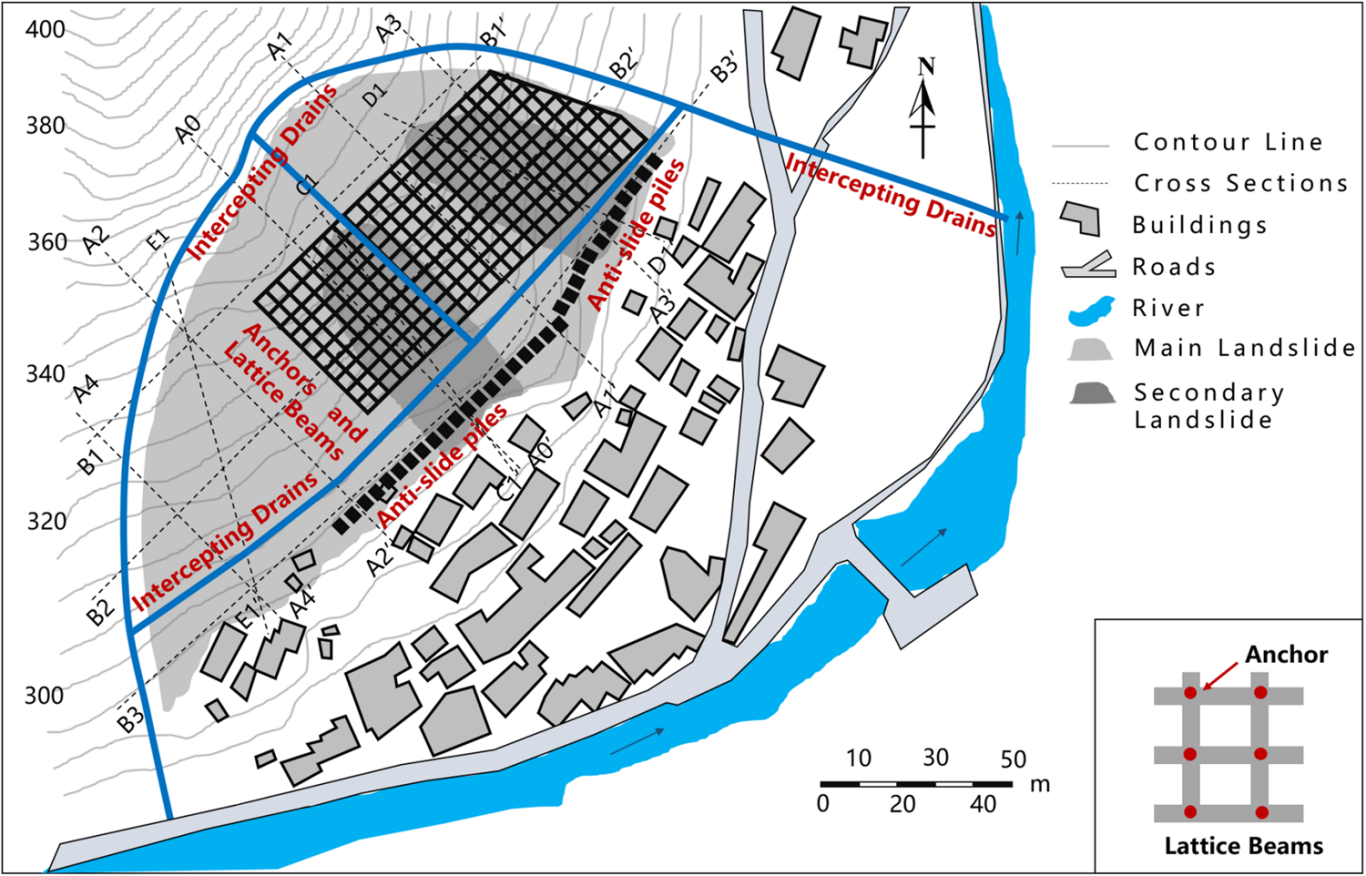
The plane layout of landslide mitigation measures (the figure was generated by Grapher 11, version 11.7.825, https://www.goldensoftware.com/products/grapher).

**Figure 7:**
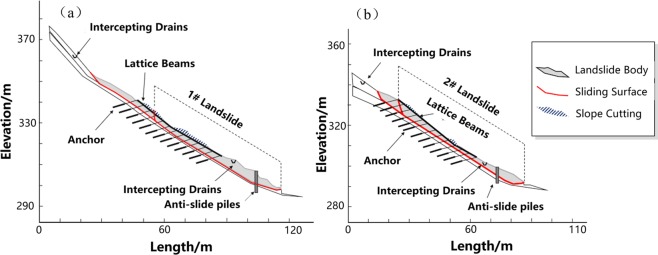
The profile layout of landslide mitigation measures. (**a**) Profile 1. (**b**) Profile 2.

The comprehensive results of stability analysis demonstrate that the slope along Profile 1 (C1-C1′) approximates the limit state, while Profile 2 (D1-D1′) is unstable. Therefore, in order to protect local buildings at the downslope area, countermeasures are required. As shown in Figs 4 and 5, the FEM analysis indicates the weak parts of the slope along both profiles. The stress and strain majorly concentrate on the toe and main body along the Profile 1, as well as the landslide crown along the Profile 2. These weak parts are supposed to subject obvious surface deformation that up towards to 142 mm as shown in Fig. 5c. In this context, we suggest structural strengthening at these weak parts, using anchor lattice beams at the landslide body, anti-slide piles at the landslide toe, as well as intercepting drains and cracks filling at the landslide crown. The length of the anchorage section is 4 m, and the anchoring force exerted by each anchor is 500 kN. The overall configuration of the landslide countermeasures is shown in Figs 6 and 7.

**Figure 8:**
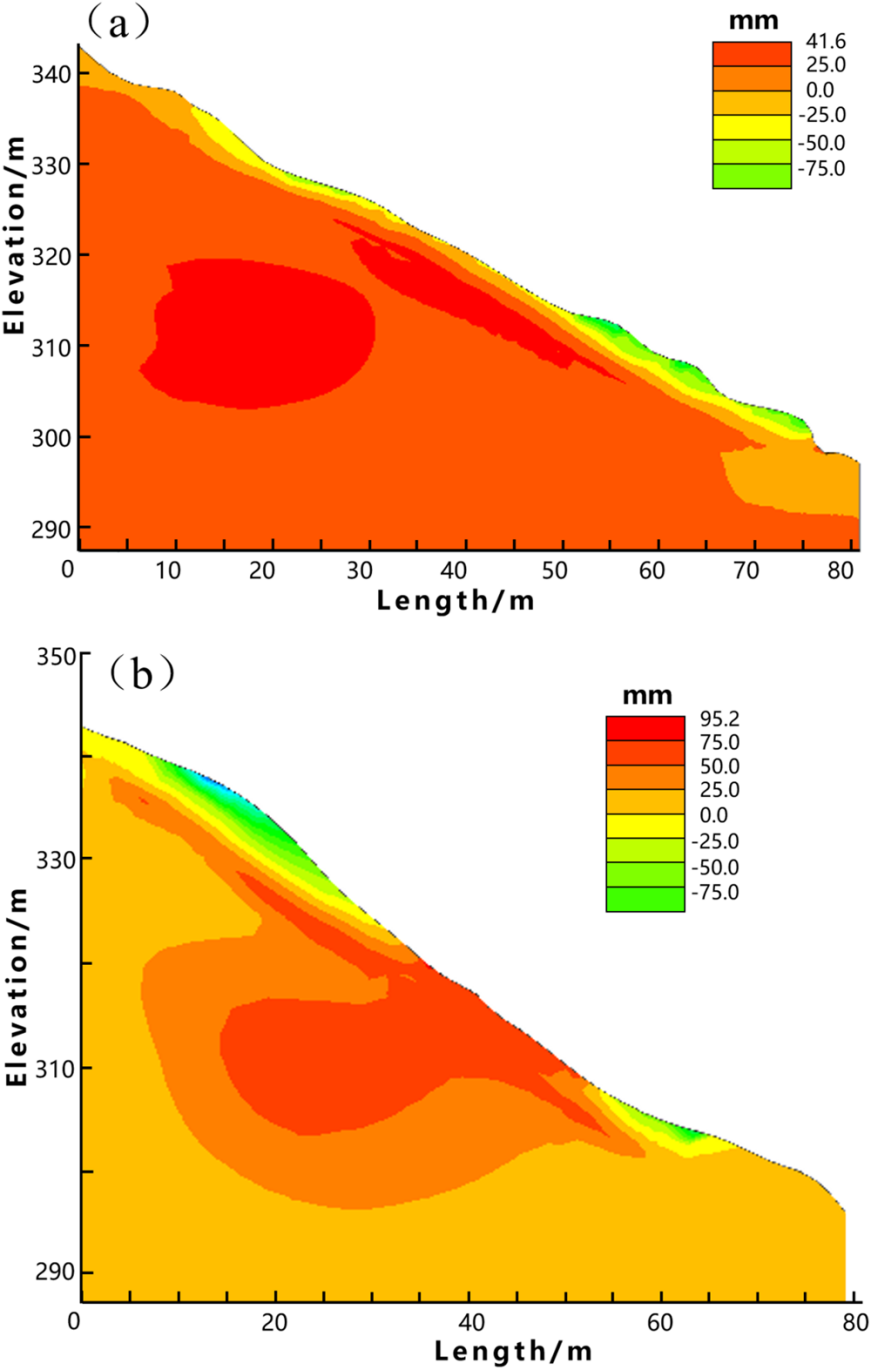
The FEM analysis results of slope deformation in x-direction (after the slope reinforcement). (**a**) Profile 1. (**b**) Profile 2.

To evaluate the effect of the countermeasures, an effect assessment is conducted in this paper by using the 2D UB method and the FEM strength reduction method. The analysis results are shown in Fig. 8 and Table 5. Figure 8 reveals an obvious control of the slope deformation after settling the structural strengthening. The maximum positive deformation in x direction of Profile 1 (C1-C1′) is reduced from 166.8 mm to 41.6 mm, while 162 mm to 95.2 mm of Profile 2 (D1-D1′). Table 5 shows that the safety factors of Profile 1 and 2 have been increased 43.6% and 43.8% respectively after the slope reinforcement. These results have demonstrated the feasibility and effectiveness of the recommended countermeasures.

**Table 5:** The analysed safety factors of the reinforced slopes.

Section	Methods		Improvement of the analyzed safety factors
	The UB method	FEM strength reduction method	
Profile 1 (along C_1_-C_1_′)	1.48	1.45	43.6%
Profile 2 (along D_1_-D_1_′)	1.32	1.28	43.8%

### Limitations

For the simplification of calculation, the reliability method introduced in this study is performed with the assumption that the shear strength parameters are independent to each other. Therefore, the negative correlation between these parameters as revealed in previous studies^55,56^, and the impact of spatial correlation of shear strength remains unknown and were not considered in this study. The above limitation should be considered in future works.

Another limitation is with respect to the FEM simulation. Presently, effects of tension cracks are not considered in our analysis. However, previous study^57^ applied the kinematic approach of limit analysis to assess the stability of uniform cohesive friction slopes with cracks, indicating that failure mechanisms departing from the crack tip can lead to a significant overestimation of the stability of the slope. For this reason, the slope profile 1 in the case study may be instable in view of many observed tension cracks in the crown. Improvement on this issue is ongoing.

The third limitation in this paper relates to the 2D simplification of the actual complex 3D slope. The composition of the slope is usually heterogeneous and, in combination with the complicated soil layers, it is commonly difficult to select appropriate profiles to analyse its stability. In the 2D limit analysis of slope stability, the slope is presumed to be infinitely wide along the out-of-plane direction, negating the 3D effects caused by the infinite width of sliding mass. In consequence, the 2D slope stability analysis may be over-conservative and insufficient. In this sense, a large and realistic 3D model should be built and analysed in order to overcome such apparent instability found in the 2D analysis.

## Conclusion

In this paper, we use a comprehensive analysis method to evaluate the stability of the Lanmuxi landslide and discuss the related countermeasures. The presented method has advantages in considering the uncertainties of the soil shear strength parameters, and generating more conservative results compared to the deterministic method.

The rational values of the sliding layer parameters are comprehensively determined by using the mean value of the back-analysis result of the reliability method, as well as the results by the deterministic method and the *in-situ* test. Following the determination of parameters, the 2D UB method of limit analysis and the FEM strength reduction method are performed for stability evaluation. The safety factors *Fs* along two typical profiles of the slope are calculated, indicating that the slopes along both profiles are unstable. The FEM-based analysis furthermore demonstrates the weak parts of the slope where stress and strain concentrate.

Structural countermeasures, using anchor lattice beams, landslide piles, and cracks filling, are suggested based on the comprehensive analysis results. Subsequently, an effect assessment based on UB method and FEM simulation is implemented. Results show notable decreasing of the deformation and about 40% increasing of the safety factors, which demonstrates the feasibility and effectiveness of the recommended countermeasures.
